# HIV Infection Disrupts the Sympatric Host–Pathogen Relationship in Human Tuberculosis

**DOI:** 10.1371/journal.pgen.1003318

**Published:** 2013-03-07

**Authors:** Lukas Fenner, Matthias Egger, Thomas Bodmer, Hansjakob Furrer, Marie Ballif, Manuel Battegay, Peter Helbling, Jan Fehr, Thomas Gsponer, Hans L. Rieder, Marcel Zwahlen, Matthias Hoffmann, Enos Bernasconi, Matthias Cavassini, Alexandra Calmy, Marisa Dolina, Reno Frei, Jean-Paul Janssens, Sonia Borrell, David Stucki, Jacques Schrenzel, Erik C. Böttger, Sebastien Gagneux, for the Swiss HIV Cohort and Molecular Epidemiology of Tuberculosis Study Groups

**Affiliations:** 1 Institute of Social and Preventive Medicine, University of Bern, Bern, Switzerland; 2 Mycobacteriology Unit, Institute for Infectious Diseases, University of Bern, Bern, Switzerland; 3 Department of Infectious Diseases, Bern University Hospital and University of Bern, Bern, Switzerland; 4 Division of Infectious Diseases and Hospital Epidemiology, University Hospital of Basel, Basel, Switzerland; 5 Division of Communicable Diseases, Federal Office of Public Health, Bern, Switzerland; 6 Division of Infectious Diseases, University Hospital Zurich, Zurich, Switzerland; 7 University of Zurich, Zurich, Switzerland; 8 Institute of Social and Preventive Medicine, University of Zurich, Zurich, Switzerland; 9 The Union, Paris, France; 10 Division of Infectious Diseases, Kantonsspital St. Gallen, St. Gallen, Switzerland; 11 Division of Infectious Diseases, Ospedale Regionale Lugano, Lugano, Switzerland; 12 Division of Infectious Diseases, University Hospital Lausanne, Lausanne, Switzerland; 13 Division of Infectious Diseases, University Hospital Geneva, Geneva, Switzerland; 14 Cantonal Institute of Microbiology, Bellinzona, Switzerland; 15 Department of Clinical Microbiology, University Hospital of Basel, Basel, Switzerland; 16 Division of Pneumology, University Hospital Geneva, Geneva, Switzerland; 17 Department of Medical Parasitology and Infection Biology, Swiss Tropical and Public Health Institute, Basel, Switzerland; 18 University of Basel, Basel, Switzerland; 19 Laboratory of Bacteriology, University Hospital of Geneva, Geneva, Switzerland; 20 Institute of Medical Microbiology, National Center for Mycobacteria, University of Zurich, Zurich, Switzerland; Georgia Institute of Technology, United States of America

## Abstract

The phylogeographic population structure of *Mycobacterium tuberculosis* suggests local adaptation to sympatric human populations. We hypothesized that HIV infection, which induces immunodeficiency, will alter the sympatric relationship between *M. tuberculosis* and its human host. To test this hypothesis, we performed a nine-year nation-wide molecular-epidemiological study of HIV–infected and HIV–negative patients with tuberculosis (TB) between 2000 and 2008 in Switzerland. We analyzed 518 TB patients of whom 112 (21.6%) were HIV–infected and 233 (45.0%) were born in Europe. We found that among European-born TB patients, recent transmission was more likely to occur in sympatric compared to allopatric host–pathogen combinations (adjusted odds ratio [OR] 7.5, 95% confidence interval [95% CI] 1.21–infinity, p = 0.03). HIV infection was significantly associated with TB caused by an allopatric (as opposed to sympatric) *M. tuberculosis* lineage (OR 7.0, 95% CI 2.5–19.1, p<0.0001). This association remained when adjusting for frequent travelling, contact with foreigners, age, sex, and country of birth (adjusted OR 5.6, 95% CI 1.5–20.8, p = 0.01). Moreover, it became stronger with greater immunosuppression as defined by CD4 T-cell depletion and was not the result of increased social mixing in HIV–infected patients. Our observation was replicated in a second independent panel of 440 *M. tuberculosis* strains collected during a population-based study in the Canton of Bern between 1991 and 2011. In summary, these findings support a model for TB in which the stable relationship between the human host and its locally adapted *M. tuberculosis* is disrupted by HIV infection.

## Author Summary

Human tuberculosis (TB) caused by *Mycobacterium tuberculosis* kills 1.5 million people each year. *M. tuberculosis* has been affecting humans for millennia, suggesting that different strain lineages may be adapted to specific human populations. The combination of a particular strain lineage and its corresponding patient population can be classified as sympatric (e.g. Euro-American lineage in Europeans) or allopatric (e.g. East-Asian lineage in Europeans). We hypothesized that infection with the human immunodeficiency virus (HIV), which impairs the human immune system, will interfere with this host–pathogen relationship. We performed a nation-wide molecular-epidemiological study of HIV–infected and HIV–negative TB patients between 2000 and 2008 in Switzerland. We found that HIV infection was associated with the less adapted allopatric lineages among patients born in Europe, and this was not explained by social or other patient factors such as increased social mixing in HIV–infected individuals. Strikingly, the association between HIV infection and less adapted *M. tuberculosis* lineages was stronger in patients with more pronounced immunodeficiency. Our observation was replicated in a second independent panel of *M. tuberculosis* strains collected during a population-based study in the Canton of Bern. In summary, our study provides evidence that the sympatric host–pathogen relationship in TB is disrupted by HIV infection.

## Introduction

Host–pathogen co-evolution plays an important role in the biology of infectious diseases [1]. Coevolution between interacting host and pathogen species is difficult to demonstrate formally, but indirect evidence can be obtained by studying geographical patterns, which can indicate local adaptation of particular pathogen variants to geographically matched host variants [2]–[4]. Local adaptation is often studied using so-called reciprocal transplant experiments, in which the fitness of locally adapted (sympatric) pathogen variants is compared to the performance of allopatric pathogen variants [2]. Studies in several invertebrate systems have shown that sympatric pathogens (infection with a phylogeographically concordant strain) tend to outperform allopatric pathogens (infection with a phylogeographically discordant strain) in the corresponding host variants [1], [5]–[7].

*Mycobacterium tuberculosis*, the agent causing human tuberculosis (TB) is an obligate human pathogen, which has been affecting humankind for millennia [8]–[13]. Contrary to previous beliefs linking the origin of TB to animal domestication ∼10,000 years ago [14], more recent data suggest that *M. tuberculosis* evolved as a human pathogen in Africa, and might have co-existed with anatomically modern humans since their origins ∼200,000 years ago [8], [10], [12], [13], [15]. Analyses of multiple global strain collections have shown that *M. tuberculosis* exhibits a phylogeographic population structure consisting of six main phylogenetic lineages associated with different geographic regions and sympatric human populations [9], [11]–[13], [16]–[20]. Studies in San Francisco, London, and Montreal have shown that these sympatric host–pathogen associations persist in cosmopolitan settings, even under a presumed degree of host and pathogen intermingling [11], [18], [19]. Moreover, transmission of *M. tuberculosis* has been shown to occur more frequently in sympatric host–pathogen combinations compared to allopatric host–pathogen combinations [9]. Taken together, these observations are consistent with the notion that the different phylogeographic lineages of *M. tuberculosis* have adapted to specific sympatric human populations [21].

Based on the assumption that *M. tuberculosis* has been coevolving with humans, and that *M. tuberculosis* has locally adapted to sympatric human populations [9], we hypothesized that HIV co-infection will alter this relationship [22]. Specifically, we postulated that because HIV induces immune suppression in humans, and because variation in host immunity will likely play a role in local adaptation, *M. tuberculosis* strains will cause disease in HIV–infected patients, irrespective of their usual sympatric host–pathogen relationship. To test this hypothesis, we performed a population-based molecular-epidemiological study of HIV–infected and HIV–negative TB patients in Switzerland between 2000 and 2008, a country with a long history of immigration [23].

## Results

### Patient characteristics and phylogeographic distribution of *M. tuberculosis* lineages

**Figure 1:**
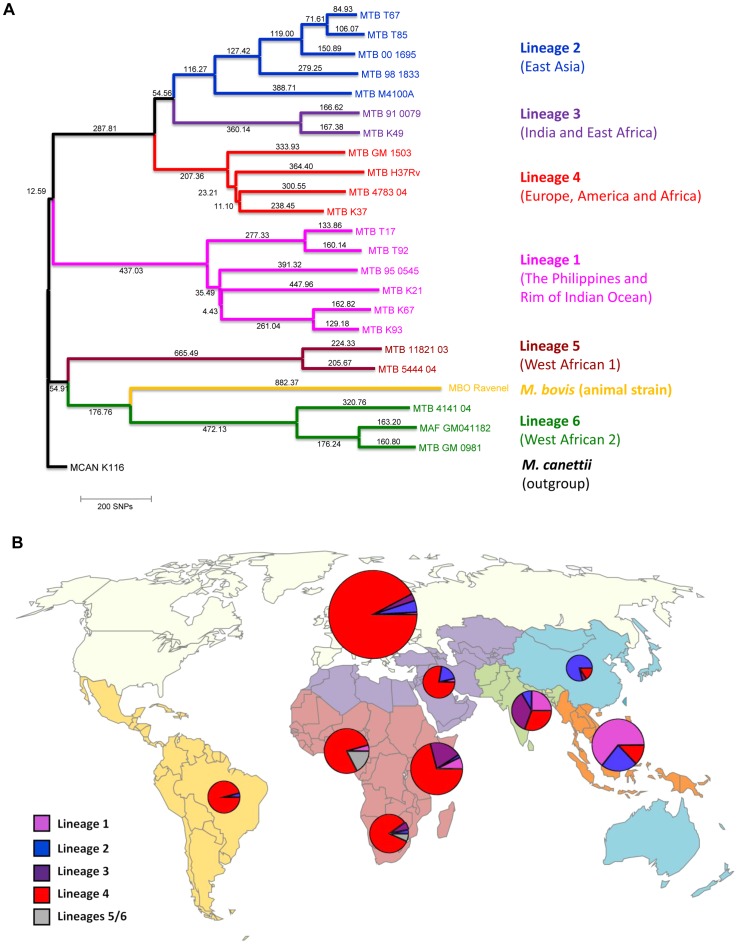
Phylogeography of the six main *Mycobacterium tuberculosis* lineages. A: Phylogenetic tree of the main *M. tuberculosis* lineages described in our study based on the neighbor-joining phylogeny across 23 *M. tuberculosis* complex whole-genome sequences (from Ref. [72]). Numbers on branches refer to the corresponding number of single nucleotide polymorphisms inferred. B: Distribution of the main phylogenetic *M. tuberculosis* lineages among Swiss tuberculosis cases included in the study (n = 518), by geographic origin of the patients. In (A) *Mycobacterium canettii* was used as the outgroup. SNPs, single nucleotide polymorphisms. In (B) the sizes of the pie charts correspond to the number of patients included in the study: European region (233 patients), Middle-East/North Africa (27), Indian subcontinent (36), Western Pacific (19), Central/South America (24), South-East Asia (48), and Eastern (55), Western (46) and Southern region (30) of sub-Saharan Africa. Lineage 1: Indo-Oceanic lineage; Lineage 2: East-Asian lineage (includes Beijing strains); Lineage 3: Delhi/CAS; Lineage 4: Euro-American lineage; Lineages 5 and 6: West African lineages.

A total of 518 patients were included in the study, of whom 112 (21.6%) were HIV–infected. Of these 518 patients, 233 (45.0%) were born in Europe (117 in Switzerland), 131 (25.3%) were born in sub-Saharan Africa, 48 (9.3%) in South-East Asia, 36 (7.0%) in the Indian subcontinent, and 24 (4.6%) in Central and South America. Similar to previous studies [9], [18], [19], we found an association between the patient's place of birth and the particular *M. tuberculosis* lineages (Figure 1). Lineage 4 (Euro-American lineage) was present in all regions, but particularly common in patients born in Europe and South America. Lineages 5 and 6 (West-African lineages also known as *M. africanum* [24]) were exclusively found in patients originating from West Africa, whereas Lineage 2 (which includes Beijing) and Lineage 1 were mainly seen in patients originating from the Western Pacific and East Asian regions. Patient characteristics are summarized in Table 1.

**Table 1:** Patient characteristics of tuberculosis (TB) patients born in Europe, by presence of allopatric and sympatric *Mycobacterium tuberculosis* strains.

Characteristic	All	Allopatric	Sympatric	*P* value
	(n = 233)	(n = 18)	(n = 215)	
Age, median (IQR), years	49 (36–71)	41.5 (32–45)	50 (37–73)	0.0029
Male sex, n (%)	126 (54.1)	10 (55.6)	116 (54.0)	0.90
Origin of birth, n (%)				0.46
Switzerland	118 (50.6)	11 (61.1)	107 (49.9)	
Europe (without Switzerland)	115 (49.4)	7 (38.9)	108 (50.2)	
Cavitary disease, n (%)	55 (23.6)	4 (22.2)	51 (23.7)	0.99^1^
Clinical manifestation, n (%)				0.54^1^
Pulmonary	191 (82.0)	16 (88.9)	175 (81.4)	
Extrapulmonary	42 (18.0)	2 (11.1)	40 (18.6)	
Recent TB within families/social surroundings	15 (6.4)	0 (0)	15 (7.0)	0.61^1^
Frequent travelling	16 (6.9)	7 (38.9)	9 (4.2)	<0.0001^1^
HIV infection, n (%)	36 (15.5)	9 (50.0)	27 (12.6)	<0.0001^1^
Immunosuppression other than HIV infection^2^, n (%)	24 (10.3)	1 (5.6)	23 (10.7)	0.70
Most likely source of HIV infection^3^, n (%)				0.94^1^
Heterosexual	15 (41.7)	5 (55.6)	10 (37.0)	
Injecting drug user	9 (25.0)	2 (22.2)	7 (25.9)	
Men having sex with men	7 (19.4)	2 (22.2)	5 (18.5)	
Others/unknown	5 (13.9)	0 (0)	5 (18.5)	

1 Fisher's exact test.

2 Use of TNF-alpha inhibitors, malignancy, organ transplantation, use of steroids, or methotrexate.

3 Among HIV–infected patients (n = 36).

95% CI, 95% confidence interval; IQR, interquartile range.

Because in European-born patients the host–pathogen combinations defined as sympatric (i.e. Lineage 4/Euro-American lineage in European-born patients) or allopatric (i.e. all other lineages in European-born patients) have been well established [9], [18], [19], [25], we focused on this patient group (n = 233) for the remaining of our analyses.

### *M. tuberculosis* transmission occurs primarily in sympatric host–pathogen combinations

*M. tuberculosis* transmission was more likely among patients in a sympatric host–pathogen relationship compared to patients in an allopatric host–pathogen relationship (adjusted odds ratio [OR] 7.5, 95% confidence interval [95% CI] 1.2-infinity, p = 0.03, Table 2). Of note, there was no molecular clustering among European-born TB patients infected with an allopatric *M. tuberculosis* strain. Moreover, we found that only the sympatric Lineage 4 (Euro-American lineage) was detected in European-born clusters as well as in mixed clusters (Table S1), suggesting that sympatric host–pathogen combinations in TB favor transmission.

**Table 2:** Recent transmission *of Mycobacterium tuberculosis* among tuberculosis (TB) cases born in the European region, according to sympatric and allopatric lineages.

Lineages	n (%) cases			Association of transmission with lineages			
	Clustered	Unclustered	*P* value^1^	OR (95% CI)	*P* value	Adjusted OR (95% CI)	*P* value
			0.024		0.048		0.027
Sympatric	42 (19.5)	173 (80.5)		6.09 (1.01-∞)		7.48 (1.21-∞)	
Allopatric	0 (0)	18 (100)		1 (Ref)	-	1 (Ref)	-

Recent transmission was determined by spoligotyping and MIRU-VNTR genotyping which is based on repetitive sequences. Clustered cases were defined as cases belonging to a molecular cluster of TB transmission based on isolates showing an identical genotyping pattern, and unclustered as cases with a unique genotyping pattern. Sympatric was defined as a strain belonging to Lineage 4 (Euro-American lineage), allopatric as a strain belonging to a lineage other than Lineage 4.

1 Fisher's exact test (1-sided).

95% CI, 95% confidence interval; ND, not defined; OR, Odds Ratio.

Odds ratios were derived from exact logistic models. Model was adjusted for age group (45 years and younger), being born in Switzerland and recent TB in families or social surroundings.

### Impact of HIV infection on the sympatric host–pathogen combination of *M. tuberculosis* among Europeans

Overall, we found that HIV infection was strongly associated with allopatric *M. tuberculosis* lineages among European-born TB patients (unadjusted OR 7.0, 95% CI 2.5–19.1, p<0.0001; Table 3). Among the allopatric lineages, we found that Lineages 1, 2 and 3 were more likely to be found in HIV–infected compared to HIV–negative patients when taking the sympatric Lineage 4 (Euro-American lineage) as the reference (Table S2). When investigating the ancestry of the nine HIV–infected patients with an allopatric *M. tuberculosis* strain, seven patients were confirmed to be of Swiss ancestry over the last three generations, and two patients had Swiss and Italian ancestors in the previous generations (Italian father in the previous generation, or emigrating from Italy in the previous generation).

**Table 3:** Unadjusted and adjusted associations between HIV infection and tuberculosis (TB) with an allopatric *Mycobacterium tuberculosis* strain among European patients (n = 233), in the context of other potential factors influencing the risk for an allopatric TB.

Variables adjusted for^1^	OR	(95% CI)	*P* value
Unadjusted	6.96	(2.54–19.08)	<0.0001
Age, sex, Swiss-born	7.54	(2.32–24.55)	0.0010
Frequent travelling	4.50	(1.49–13.61)	0.0080
Age, sex, Swiss-Born, frequent travelling, contact with foreign-born population	5.57	(1.49–20.81)	0.011
Immunosuppression^2^	7.06	(2.57–19.42)	<0.0001
Age, sex, Swiss-born, frequent travelling, contact with foreign-born population, immunosuppression^2^	5.51	(1.47–20.61)	0.011

HIV–negative TB patients were used as the reference group.

**Figure 2:**
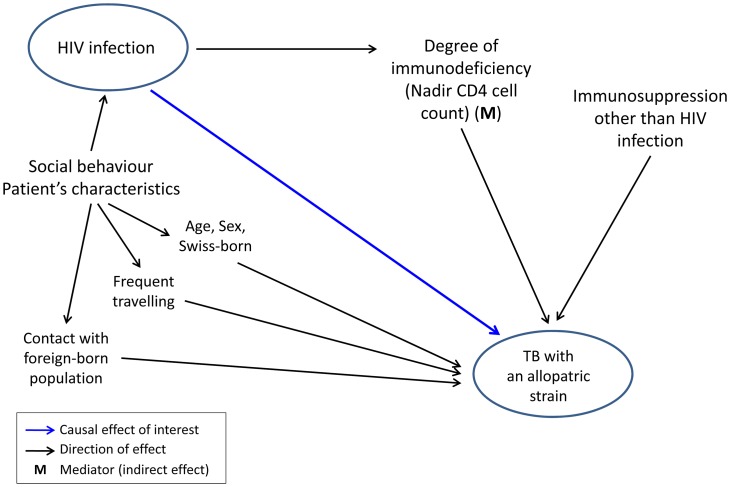
Graphical model showing direct and indirect potential effects of HIV infection on tuberculosis (TB) caused by an allopatric *Mycobacterium tuberculosis* strain, in the context of other potential factors influencing this association.

1 See Figure 2 for a graphical overview of associations.

2 Immunosuppression other than HIV infection (use of anti-TNF blockers, malignancy, organ transplantation, use of steroids or methotrexate).

OR, odds ratio; 95% CI, 95% confidence interval.

Several factors could contribute to the association between HIV infection and allopatric lineages. We found that patients with an allopatric *M. tuberculosis* lineage were younger (median age 41.5 versus 50 years), and had more often a history of frequent travelling (38.9% versus 4.2%, p<0.0001). Therefore, we developed a model (Figure 2) to take these and other putative confounding variables into account. These variables included age, sex, country of birth, frequent travelling, contact with the foreign-born population, and non–HIV associated immunosuppression. We considered “TB with an allopatric strain” as the outcome because disease is the only measurable outcome with a sympatric or allopatric *M. tuberculosis* strain (only diseased individuals can yield a positive mycobacterial culture). Our multivariate analyses revealed that the association between HIV infection and allopatry remained statistically significant after adjustment for all social and patient factors included in our model (OR 5.5, 95% CI 1.5–20.6, p = 0.01, Table 3). Age, sex, being Swiss-born, and non–HIV associated immunosuppression had only a minor effect on the association between HIV infection and TB with an allopatric strain (Table 3). In contrast, a history of repeated travelling to low-income countries had a stronger effect, decreasing the OR to 4.50 (95% CI 1.5–13.6, p = 0.008, Table 3) when adjusting for this variable.

### Impact of HIV infection on the sympatric host–pathogen association is a function of the degree of HIV–induced immunosuppression

We also tested if the degree of immunodeficiency as measured by the nadir CD4 T cell count (defined as the lowest CD4 T cell count ever measured in a patient) would have an impact on the association between host population and *M. tuberculosis* lineage. Among Europeans, the strength of the association between HIV infection and allopatric lineages increased with a decreasing nadir CD4 T cell count in a dose-dependent manner: from an OR of 4.6 (95% CI 0.9–24.7) in patients with a nadir CD4 T cell count of >200 cells/µL to an OR of 12.5 (95% CI 2.6–60.8) in patients with nadir CD4 T cell counts <50 cells/µL (test for trend p<0.0001; HIV–negative patients as reference). This trend remained statistically significant when adjusting for age, sex, being born in Switzerland, frequent travelling, contact with the foreign-born population, and non–HIV associated immunosuppression (Table 4).

**Table 4:** Association between the degree of immunodeficiency and tuberculosis with an allopatric *Mycobacterium tuberculosis* strain among European patients (n = 233).

Degree of immunodeficiency	n	Unadjusted			Adjusted		
		OR	95% OR	*P* value	OR	95% OR	*P* value
Nadir CD4 T cell count (CD4 cells/µl)				<0.0001			0.0050
HIV–negative	197	1.0	(ref)		1.0	(ref)	
≥200	11	4.64	(0.87–24.70)		2.56	(0.37–17.65)	
50–199	17	6.43	(1.74–23.70)		6.96	(1.18–41.11)	
<50	8	12.53	(2.58–60.84)		13.0	(1.54–109.75)	

Model was adjusted for age, sex, Swiss-born, frequent travelling, contact with foreign-born population, and immunosuppression other than HIV infection (see Figure 2 for a graphical overview).

*P* values of linear tests for trend are shown.

### Impact of social mixing on the sympatric host–pathogen association

Increased contact with foreigners originating from high TB burden countries, who have a higher risk of TB [26] and are more likely to have TB caused by an allopatric *M. tuberculosis* strain, could also lead to an allopatric host–pathogen relationship in European-born patients. However, the association between HIV and allopatry remained statistically significant when adjusting for this variable (Table 3). Furthermore, we examined molecular clusters defined by standard bacterial genotyping [27], [28], to test the hypothesis that HIV–infected patients were more frequently seen among ethnically mixed clusters where transmission occurred between non-European and European-born cases [29]. We found that the prevalence of HIV infection was similar among TB cases in mixed clusters (5 HIV–infected cases out of 26 cases, 19.2%) and among cases in clusters involving only European-born cases (4 out of 26 cases, 15.4%, see Table S1).

### Sensitivity analyses

When restricting the main analysis (n = 233) to European-born patients without a history of frequent travelling, we found that the association between HIV infection and allopatric TB remained statistically significant (adjusted OR 6.96, 95% CI 1.25–38.88, p = 0.027). Furthermore, we explored associations of socio-demographic and clinical factors with TB with an allopatric *M. tuberculosis* strain in a model focusing on HIV–infected European patients only (Figure S1, Table S3): frequent travelling was confirmed to be an important factor, and patients with a low nadir CD4 T cell count tended to be associated with an allopatric TB although the associations did not reach statistical significance (Table S3). Finally, we obtained very similar results for the association between HIV infection and allopatric TB (Table S4), and for the association between the degree of immunodeficiency and allopatric TB (Table S5) when repeating analyses using a Bayesian approach [30], which is more robust when numbers are small.

### Other supporting information

The birth origin of HIV–infected and non-infected patients is shown on a map in Figure S2. The main phylogenetic *M. tuberculosis* lineages stratified by place of birth and HIV status are presented in Table S6.

### Replication in a second panel of *M. tuberculosis* strains

To replicate our main finding, we investigated a second panel of *M. tuberculosis* strains from an ongoing population-based TB study in the Canton of Bern, Switzerland, between 1991 and 2011. Of the 1,642 *M. tuberculosis* isolates analyzed, 1,350 (82.2%) belonged to Lineage 4 (Euro-American lineage), and 292 (17.8%) to non-Euro-American lineages (Lineages 1, 2, 3, 5 or 6). We compared all 40 European-born patients with an allopatric strain (non-Lineage 4) with 400 randomly selected European-born patients with a sympatric strain (Lineage 4). We found that the proportion of HIV infection was 4.5 (95% CI 1.6–11.9) times higher in patients with an allopatric strain compared to patients with a sympatric strain (12.5% versus 2.8%, p = 0.010, Table 5).

**Table 5:** HIV status in tuberculosis (TB) patients with an allopatric compared to patients with a sympatric *Mycobacterium tuberculosis* strain among European patients in a second panel.

HIV status	TB cases, n (%)			*P* value
	All(n = 440)	Allopatric(n = 40)	Sympatric(n = 400)	
				0.010^1^
HIV–infected	16 (3.6)	5 (12.5)	11 (2.7)	
HIV–negative	424 (96.4)	35 (87.5)	389 (97.3)	
Prevalence ratio (95% CI)		4.55 (1.66–12.43)		

Patient isolates were obtained from a population-based TB study (n = 1,642) in the Canton of Bern, Switzerland, diagnosed between 1991 and 2011.

Sympatric was defined as a strain belonging to Lineage 4 (Euro-American lineage), allopatric as a strain belonging to a lineage other than Lineage 4.

1 Fisher's exact test.

95% CI, 95% confidence interval.

## Discussion

The phylogeographic distribution of *M. tuberculosis* lineages observed here suggests local adaptation to sympatric human populations. In contrast, we found that allopatric host–pathogen relationships in European-born TB patients were strongly associated with HIV co-infection. The association with HIV infection became stronger in a ‘dose-dependent’ manner in patients with a history of more pronounced immunodeficiency, and was not explained only by frequent travelling to high TB-incidence countries or increased social mixing with the foreign-born population. The association of *M. tuberculosis* lineages with sympatric patient populations reported here is in agreement with previous findings [9], [11]–[13], [16]–[19]. Similarly, our finding that recent TB transmission was more likely to occur in sympatric compared to allopatric host–pathogen combinations supports previous work [9]. Taken together, these data are consistent with local adaptation of *M. tuberculosis* to different human populations, which in turn can be viewed as indirect evidence for coevolution between *M. tuberculosis* and its human host [1]–[4], [9]–[13].

We found that TB allopatric host–pathogen combinations were strongly associated with HIV infection in a nation-wide study and a second panel of strains from one Canton of Switzerland. This supports the notion that *M. tuberculosis* lineages have evolved subtle differences in their interaction with different human immune systems. However, in the presence of HIV–induced immunodeficiency, any *M. tuberculosis* lineage seems to cause disease in a given human host. *M. tuberculosis* is an obligate human pathogen which lives in constant interaction with the host immune system [31]. Human populations, however, are known to differ genetically and immunologically [15]. The clinical disease reflects host-dependent immune-pathological processes [31]. In other words, while initially triggered by the pathogen, it is the host immune response which is ultimately responsible for the chronic inflammation and associated tissue destructions. These processes contribute to the successful transmission of *M. tuberculosis* [22], [32]. On the other hand, only 5–10% of the 2 billion individuals estimated to be latently infected with *M. tuberculosis* globally will develop active TB during their lifetime [33]–[35]. Hence most of the time, humans are able to control the infection. In our study, we chose culture-confirmed TB cases as the main endpoint which reflects successful transmission and progression from infection to active disease.

Our study on the association between allopatric TB and HIV was able to control for important cofactors [36], [37]. These cofactors included frequent travelling abroad and increased contact to foreign-born populations. A particularly important cofactor for allopatric TB was frequent travelling to high TB burden countries with potential exposure to foreign *M. tuberculosis* strains; HIV–infected individuals may be at a higher risk for travel-related infectious diseases [38]. However, the association between HIV infection and allopatric TB remained even when adjusting for these behavioral and other patient characteristics. A previous study reporting on allopatric TB and HIV was not able to control for these factors [9]. Furthermore, we found no evidence for increased social mixing among HIV–infected individuals, which argues against mere social factors leading to the association between allopatric TB and HIV.

A biological basis for this association is further supported by the striking dose-dependency we observed with increasing immunosuppression as defined by lower nadir CD4 T cell counts. Of note, this trend was also independent of other variables. Low nadir CD4 T cell counts are associated with incomplete immune recovery after starting combination antiretroviral therapy [39], [40] and impaired functional immune restoration despite normalization of CD4 T cells [41]. More generally, infection with HIV and *M. tuberculosis* interferes with the immune system in many ways [42], [43]. HIV infection disrupts the function of *M. tuberculosis*-infected macrophages [44], [45], but also seems to reduce the number and functionality of *M. tuberculosis*-specific T cells over time [46]. On the other hand, *M. tuberculosis* strains have been shown to induce variable immune responses [47]. Based on these observations, it is reasonable to hypothesize that HIV/TB co-infection might impact immune cell functions, intracellular signaling and immune regulation, perhaps leading to an immune response less capable of discriminating between *M. tuberculosis* variants.

Besides *M. tuberculosis*, several other human pathogenic bacteria exhibit phylogeographic population structures, possibly reflecting local adaptation to different human populations. These include *Haemophilus influenzae* [48], *Streptococcus mutans* [49], *M. leprae* [50] and *Helicobacter pylori* [51], [52]. Interestingly, like *M. tuberculosis*, all of these microbes are obligate human pathogens. In the case of *H. pylori*, functional studies have shown that strains associated with South America have adapted their adhesins to the human blood group O, which is particularly frequent in native populations of this region [53]. Similarly, a study of the bacterial genome evolution of an asymptomatic *Escherichia coli* bacteriuria strain showed adaptation at the genomic level in distinct human hosts [54]. No similar experimental work has yet been carried out in TB. However, several studies have reported associations between human genetic polymorphisms and particular *M. tuberculosis* lineages [55]–[59], indicating possible interaction between human and *M. tuberculosis* variation. Whether such variation in pathogen and host genetics can be attributed to co-evolution will be difficult to demonstrate conclusively, but the data presented here support this possibility.

The strength of our study was that we used a nation-wide sample to specifically look at the impact of HIV infection on the host–pathogen relationship in human TB. Yet, our study is limited by the relatively small sample size, and the difficulty to quantify the complex social context through which the host–pathogen relationship is influenced in human TB. In addition, we looked at European-born patients only, because sympatric and allopatric host–pathogen combinations are more easily defined for this patient population [9], [18], [19], [25]. Additional studies in large cosmopolitan cities of Asia and Africa would be required to test whether the association between allopatric TB and HIV holds true in these settings. Ultimately, detailed experimental work is needed to establish the biological basis of the host–pathogen association in human TB.

In conclusion, our data suggest that the phylogeographical host–pathogen relationship in TB influences transmission patterns. Among the studied European-born TB patients, we showed that HIV infection disrupts the sympatric host–pathogen relationship in human TB, and that this effect increased as a function of immunodeficiency. Various interactions between HIV and *M. tuberculosis* at the cellular level make an association biologically plausible [42], [43]. Further studies are needed to investigate the impact of HIV on the genetic population structure of *M. tuberculosis* with its consequences for transmission and clinical manifestations in high TB burden countries [36]. This will lead to a better understanding of biological factors that shape the current HIV/TB syndemic [60].

## Methods

### Study setting

The Swiss Molecular Epidemiology of Tuberculosis (SMET) study is a collaborative project between the Swiss HIV Cohort Study (SHCS), the National Center for Mycobacteria, diagnostic microbiology laboratories, departments of respiratory medicine and public health, and the Federal Office of Public Health (FOPH) [29], [61], [62]. The overarching aims were to examine the genetic population structure of *M. tuberculosis* and the associations between strain variation, patient origin, and clinical characteristics in HIV–infected and HIV–negative TB patients in Switzerland. Further information on the SMET project is available at www.tb-network.ch. All participating sites are listed in the Acknowledgements.

The SHCS is a prospective observational study of HIV–infected individuals followed up in HIV outpatient clinics in Switzerland [63]. All HIV–infected patients diagnosed with TB between 2000 and 2008 whose *M. tuberculosis* complex (MTBC) isolate was available were included in the SMET study [29]. Furthermore, we randomly selected 288 from the 4,221 culture-confirmed TB cases reported to the National TB Surveillance Registry during the same period (approximately three cases for one HIV–infected TB case within the SHCS). Finally, all reported drug-resistant TB cases were included. Two *M. bovis* isolates were excluded from this study as they are animal-adapted species within the MTBC and therefore represent a different host–pathogen relationship.

### Clinical data collection and definitions

We obtained clinical data by standardized questionnaires sent to the treating physicians and extracted relevant data from the SHCS database. We collected socio-demographic data (age, sex, origin of birth, citizenship, legal status, immunosuppressive therapy, risk factors for TB such as recent TB within family or immediate social surroundings in the last two years), laboratory parameters (CD4 cell count and plasma HIV RNA in HIV–infected cases) and clinical information (site of disease, radiography findings). Chest radiography parameters were consolidation, cavitations, enlarged intrathoracic lymph nodes and pleural thickening. Any drug resistance was defined as any resistance to isoniazid, rifampicin or ethambutol as reported to the FOPH. Most TB cases in Switzerland are treated under the guidance of experienced infectious and respiratory disease specialists, and the clinical data were of high quality.

Geographic origin of patients was defined as the country of birth, and countries were grouped in seven geographic regions (see Figure 1) according to the current understanding of the phylogeography of *M. tuberculosis* [25]. Birth country was used as a proxy of the ancestry of the study population. Immunosuppression due to other causes than HIV infection was defined as use of TNF-alpha inhibitors, malignancy, solid organ transplantation, use of steroids or methotrexate. Nadir CD4 T cell count was defined as the lowest CD4 T cell count (cells/µL) ever measured in a patient. Nadir CD4 T cell count is a predictor of poor immune recovery after ART [39]. Travel history was extracted from the free text field “Risk factor for TB” and defined as repeated travelling of longer duration (>30 days) to low-income countries with a high TB burden and a relevant exposure to *M. tuberculosis* according to the physician's judgment. Belonging to a molecular cluster involving Swiss-born and foreign-born TB cases was used as a proxy for contact with the foreign-born population.

### Molecular analyses

Mycobacterial isolates were cultured and DNA extracted according to standard laboratory procedures. We used spacer oligonucleotide typing (spoligotyping) and 24-loci mycobacterial interspersed repetitive units (MIRU-VNTR) which are based on repetitive DNA sequences as genotyping tools with high discriminatory power to identify recent TB transmission [29], [64]–[66]. Data were analyzed with the MIRU-VNTRplus online tool (http://www.miru-vntrplus.org). Molecular clusters were defined as a group of completely identical isolates in the spoligotyping and MIRU-VNTR pattern indicating a chain of TB transmission. In addition, we used single nucleotide polymorphisms (SNPs) as stable genetic markers to define the main phylogenetic *M. tuberculosis* lineages [67]. Lineages were determined by SNPs using multiplex real-time PCR with fluorescence-labeled probes (Taqman, Applied Biosystems, USA) adopted from previous studies [9], [12], [67], [68]. The SNP used to define Lineage 4 was originally described by Sreevatsan et al. [69] and shown to be specific to this lineage [9].

### Graphical models

Graphical models were built using the principles of directed acyclic graphs [70]. Our model considered infection and disease as a combined outcome (“TB with an allopatric strain”). Our hypothesis that HIV infection causes TB with an allopatric strain is shown as a potentially causal direct effect, and risk factors potentially influencing this effect are shown in the hypothetical direction. Mediators represent variables that are caused by the independent variable and, in turn, have a direct effect on the outcome variable. We included age and sex in our model as risk factors for infection and disease [37]. We also considered contact with the foreign-born population who have a higher risk for TB compared to the native Swiss population [26] and who have a higher risk of exposure to “foreign” *M. tuberculosis* strains. Finally, we included frequent traveling to countries with a high TB burden, which increases exposure risk and thus potentially infection risk with “foreign” *M. tuberculosis* strains (Figure 2).

### Statistical analyses

We used χ2 tests or Fisher's exact tests to assess differences between groups in binary variables, and the Wilcoxon rank sum test for continuous variables (Table 1, Table 2). Univariate and multivariate exact logistic regression models were fitted to estimate the association between transmission as defined by molecular clustering and patients with sympatric *M. tuberculosis* lineages (patients with allopatric lineages were used as the reference, Table 2). Results were presented as ORs unadjusted and adjusted for age group, being born in Switzerland and recent TB in families or social surroundings. To assess the association of HIV infection with allopatric TB, we fitted univariate and multivariate logistic models (Table 3), and presented ORs unadjusted and adjusted for age, sex, Swiss-born, frequent travelling, contact with foreign born populations, and/or immunosuppression. We used univariate and multivariate logistic models to estimate the association between the degree of immunodeficiency and allopatric TB (Table 4), and presented ORs unadjusted and adjusted for age, sex, Swiss-born, frequent travelling, contact with foreign-born populations, and immunosuppression other than HIV infection. Finally, we determined statistical significance of HIV prevalence in patients with allopatric *M. tuberculosis* lineages compared to patients with sympatric lineages using Fisher's exact tests (Table 5). All analyses were performed in Stata version 11.1 (Stata Corporation, College Station, TX, USA).

### Sensitivity analyses

In sensitivity analyses, we excluded patients with a history of frequent travelling to remove its influence on the association between HIV infection and allopatric lineages. In addition, we repeated the analyses using fully probabilistic Bayesian methods using weakly informative prior distributions [71]. The CIs reported from these analyses are 95% credible intervals and correspond to tail probabilities of the coefficient's posterior distributions. Bayesian statistics are less sensitive to errors when calculating estimators and CIs in small datasets.

### Second panel of *M. tuberculosis* strains

We obtained 1,642 *M. tuberculosis* isolates from all TB cases (n = 1,940, 84.6%) notified in the Canton of Bern, Switzerland, between 1991 and 2011. For all patient isolates, we determined the main phylogenetic *M. tuberculosis* lineages. Of these, we included all patient isolates belonging to a non-Euro-American lineage (Lineage 1, 2, 3, 5 or 6) from European-born TB patients (40 of a total of 292 isolates belonging to lineages other than Lineage 4). Furthermore, we randomly selected control strains belonging to the Euro-American lineage (Lineage 4) from European-born TB patients (400 of a total of 1,350 isolates belonging to Lineage 4). European ancestry was confirmed in HIV–infected patients with an allopatric *M. tuberculosis* strain. Finally, we determined the HIV status in these patients using the same procedures as in the main sample.

### Ethics approval

The study was approved by the ethics committee of the Canton of Bern, Switzerland. Written informed consent was obtained from all patients enrolled in the SHCS. For patients outside the SHCS, written informed consent was obtained by the treating physicians. In some cases informed consent could not be obtained from the patient because he or she could not be located or was known to have died. For these cases we obtained permission from the Federal expert commission on confidentiality in medical research to use the data provided by the treating physician.

## Supporting Information

Figure S1 Graphical model showing direct potential effects on tuberculosis (TB) with an allopatric *Mycobacterium tuberculosis* strain among HIV–infected patients.
(PDF)

Figure S2 Distribution of tuberculosis (TB) cases included in the study, by origin of birth and HIV status.
(PDF)

Table S1 Distribution of the main phylogenetic *Mycobacterium tuberculosis* lineages according to molecular clusters involving either non-European-born tuberculosis (TB) cases only, European-born cases, or mixed clusters involving both non-European-born and European-born TB cases.
(PDF)

Table S2 Crude and adjusted analysis comparing HIV–infected and HIV–negative tuberculosis (TB) patients born in Europe (n = 233) across the four most frequent *Mycobacterium tuberculosis* lineages.
(PDF)

Table S3 Associations of socio-demographic and clinical factors with tuberculosis (TB) with an allopatric *Mycobacterium tuberculosis* strain among HIV–infected European patients (n = 36).
(PDF)

Table S4 Associations between HIV infection and tuberculosis (TB) with an allopatric *Mycobacterium tuberculosis* strain among European patients (n = 233) in the context of other potential factors influencing this association, using Bayesian statistics and presented as unadjusted or adjusted odds ratios.
(PDF)

Table S5 Association between the degree of immunodeficiency and tuberculosis (TB) with an allopatric *Mycobacterium tuberculosis* among European patients (n = 233) using Bayesian statistics.
(PDF)

Table S6 Comparing the main phylogenetic *Mycobacterium tuberculosis* lineages, by HIV status and birth region.
(PDF)
