# Supplementary material for: HIV Infection Disrupts the Sympatric Host–Pathogen Relationship in Human Tuberculosis
Source: PLoS Genet. 2013 Mar 7;9(3):e1003318. doi: 10.1371/journal.pgen.1003318 (PMC3591267; doi:10.1371/journal.pgen.1003318)

Figure S1. Graphical model showing direct potential effects on tuberculosis (TB) with an allopatric *Mycobacterium tuberculosis* strain among HIV-infected patients.

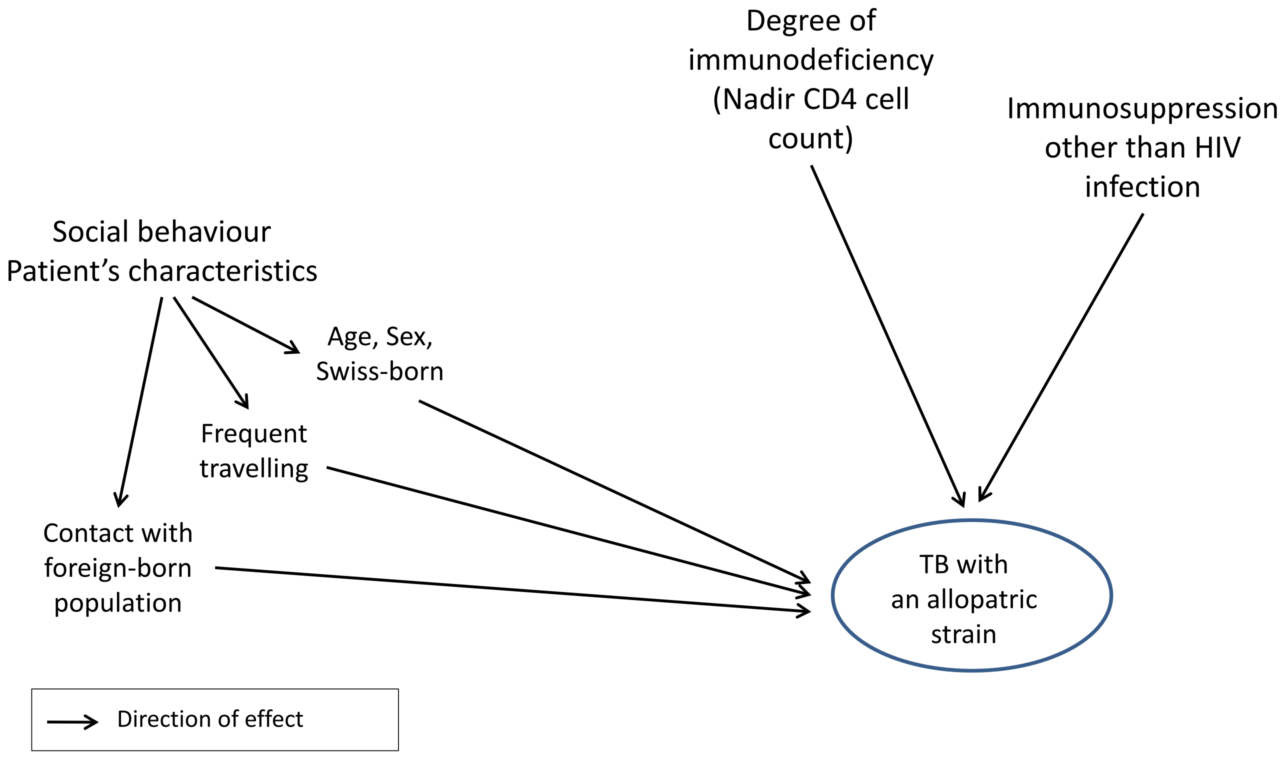

Supplement: Figure S1 — Graphical model showing direct potential effects on tuberculosis (TB) with an allopatric Mycobacterium tuberculosis strain among HIV–infected patients. (PDF) [file pgen.1003318.s001.pdf]
