# Supplementary material for: HIV Infection Disrupts the Sympatric Host–Pathogen Relationship in Human Tuberculosis
Source: PLoS Genet. 2013 Mar 7;9(3):e1003318. doi: 10.1371/journal.pgen.1003318 (PMC3591267; doi:10.1371/journal.pgen.1003318)

Figure S2. Distribution of tuberculosis (TB) cases included in the study, by place of birth and HIV status. +, HIV-infected TB cases; -, HIV-negative TB cases.

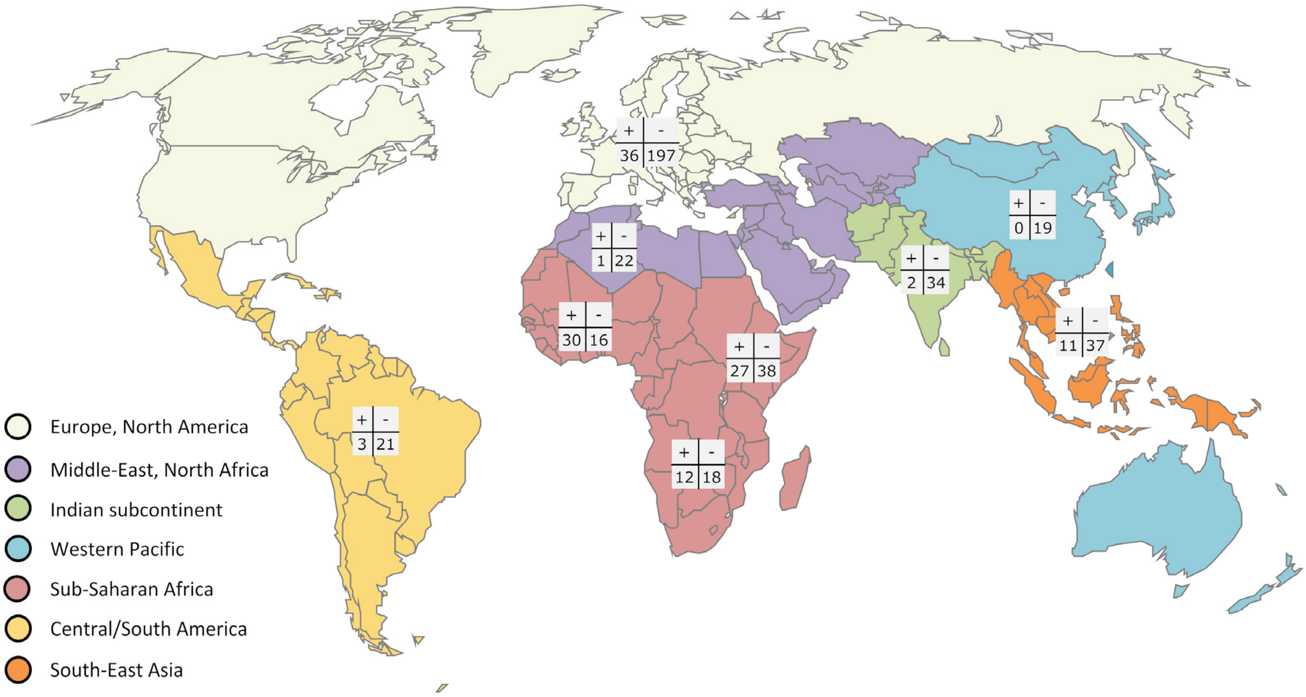

Supplement: Figure S2 — Distribution of tuberculosis (TB) cases included in the study, by origin of birth and HIV status. (PDF) [file pgen.1003318.s002.pdf]
