# Supplementary material for: HIV Infection Disrupts the Sympatric Host–Pathogen Relationship in Human Tuberculosis
Source: PLoS Genet. 2013 Mar 7;9(3):e1003318. doi: 10.1371/journal.pgen.1003318 (PMC3591267; doi:10.1371/journal.pgen.1003318)
Supplement: Table S1 — Distribution of the main phylogenetic Mycobacterium tuberculosis lineages according to molecular clusters involving either non-European-born tuberculosis (TB) cases only, European-born cases, or mixed clusters involving both non-European-born and European-born TB cases. (PDF) [file pgen.1003318.s003.pdf]

Table S1. Distribution of the main phylogenetic *Mycobacterium tuberculosis* lineages according to molecular clusters involving either non-European-born tuberculosis (TB) cases only, European-born TB cases only, or mixed clusters involving both non-European-born and European-born TB cases.

Cluster category	No. of clusters	No. of patients	No. of patients (%) [1]				
			Lineage 1	Lineage 2	Lineage 3	Lineage 4	HIV-infected [2]
Non-EU-born only	16	38	5 (13.2)	6 (15.8)	5 (13.2)	22 (57.9)	12 (31.6)
Mixed	8	26	0	0	0	26 (100)	5 (19.2)
EU-born only	11	26	0	0	0	26 (100)	4 (15.4)

P values by Fisher's exact test: [1] $p<0.0001$ across lineages and cluster categories; [2] $p=0.34$ across HIV status and cluster categories

EU, European

Lineage 1: Indo-Oceanic lineage; Lineage 2: East-Asian lineage (includes Beijing strains); Lineage 3: Delhi/CAS; Lineage 4: Euro-American lineage
