# Supplementary material for: HIV Infection Disrupts the Sympatric Host–Pathogen Relationship in Human Tuberculosis
Source: PLoS Genet. 2013 Mar 7;9(3):e1003318. doi: 10.1371/journal.pgen.1003318 (PMC3591267; doi:10.1371/journal.pgen.1003318)
Supplement: Table S2 — Crude and adjusted analysis comparing HIV–infected and HIV–negative tuberculosis (TB) patients born in Europe (n = 233) across the four most frequent Mycobacterium tuberculosis lineages. (PDF) [file pgen.1003318.s004.pdf]

Table S2. Crude and adjusted analysis comparing HIV-infected and HIV-negative tuberculosis patients born in Europe (n=233) across the four most frequent *Mycobacterium tuberculosis* lineages.

**Main lineage**	**Unadjusted**			**Adjusted**		
	**OR**	**(95% CI)**	***P* value**	**OR**	**(95% CI)**	***P* value**
Lineage 1	7.0	(0.42-114.6)	0.17	6.15	(0.30-125.53)	0.24
Lineage 2	4.6	(1.23-17.51)	0.023	3.22	(0.80-13.04)	0.1
Lineage 3	13.93	(2.43-79.71)	0.003	12.54	(2.04-77.21)	0.006
Lineage 4	1	(ref)	-	1	(ref)	-

95% CI, 95% confidence interval; OR, Odds Ratio

Model was adjusted for age, sex, and being born in Switzerland

Lineage 1: Indo-Oceanic lineage; Lineage 2: East-Asian lineage (includes Beijing strains); Lineage 3: Delhi/CAS; Lineage 4: Euro-American lineage
