# Supplementary material for: HIV Infection Disrupts the Sympatric Host–Pathogen Relationship in Human Tuberculosis
Source: PLoS Genet. 2013 Mar 7;9(3):e1003318. doi: 10.1371/journal.pgen.1003318 (PMC3591267; doi:10.1371/journal.pgen.1003318)
Supplement: Table S3 — Associations of socio-demographic and clinical factors with tuberculosis (TB) with an allopatric Mycobacterium tuberculosis strain among HIV–infected European patients (n = 36). (PDF) [file pgen.1003318.s005.pdf]

Table S3. Associations of sociodemographic and clinical factors with tuberculosis with an allopatric *Mycobacterium tuberculosis* strain among HIV-infected European patients (n=36).

**Variables adjusted for** [1]	**OR**	**(95% CI)**	***P* value**
Male sex	0.85	(0.17-4.17)	0.84
Age groups, years			0.29
16-49		1.0 (ref)	
≥50	0.54	(0.18-1.67)	
Swiss-born	6.64	(0.90-∞)	0.066
Frequent travelling	10.0	(1.69-59.31)	0.011
Immunosuppression [3]	1.91	(0.21-∞)	0.60
Contact with foreign-born			0.43 [2]
No or unknown contact		1.0 (ref)	
Foreign contact	2.56	(0-99.67)	
Swiss contact	0.50	(0-4.47)	
Degree of immunodeficiency (nadir CD4 cell count cells/µl)			0.36 [2]
≥200		1.0 (ref)	
50-199	1.38	(0.21-9.24)	
<50	2.70	(0.33-21.98)	

[1] See Figure S2 for a graphical overview

[2] *P* values of linear tests for trend

[3] Immunosuppression other than HIV infection (use of anti-TNF blockers, malignancy, organ transplantation, use of steroids or methotrexate)

OR, odds ratio; 95% CI, 95% confidence interval
