# Supplementary material for: HIV Infection Disrupts the Sympatric Host–Pathogen Relationship in Human Tuberculosis
Source: PLoS Genet. 2013 Mar 7;9(3):e1003318. doi: 10.1371/journal.pgen.1003318 (PMC3591267; doi:10.1371/journal.pgen.1003318)
Supplement: Table S4 — Associations between HIV infection and tuberculosis (TB) with an allopatric Mycobacterium tuberculosis strain among European patients (n = 233) in the context of other potential factors influencing this association, using Bayesian statistics and presented as unadjusted or adjusted odds ratios. (PDF) [file pgen.1003318.s006.pdf]

Table S4. Associations between HIV infection and tuberculosis with an allopatric *Mycobacterium tuberculosis* strain among European patients (n=233) in the context of other potential factors influencing this association, using Bayesian statistics and presented as unadjusted or adjusted odds ratios.

**Variables adjusted for** [1]	**OR**	**(95% CI)**	***P* value**
Unadjusted	6.14	(2.32-16.24)	<0.0001
Age, sex, Swiss-born	6.06	(2.06-17.79)	0.0010
Frequent travelling	4.07	(1.42-11.66)	<0.0001
Immunosuppression [2]	6.20	(2.34-16.43)	<0.0001
Age, sex, Swiss-Born, frequent travelling, contact with foreign-born population	4.33	(1.45-13.91)	0.014
Immunosuppression [2]	6.20	(2.34-16.43)	<0.0001
Age, sex, Swiss-born, frequent travelling, contact with foreign-born population, immunosuppression [2]	4.31	(1.34-13.87)	0.014

[1] See Figure 2 for a graphical overview

[2] Immunosuppression other than HIV infection (use of anti-TNF blockers, malignancy, organ transplantation, use of steroids or methotrexate)

OR, odds ratio; 95% CI, 95% confidence interval
