# Supplementary material for: HIV Infection Disrupts the Sympatric Host–Pathogen Relationship in Human Tuberculosis
Source: PLoS Genet. 2013 Mar 7;9(3):e1003318. doi: 10.1371/journal.pgen.1003318 (PMC3591267; doi:10.1371/journal.pgen.1003318)
Supplement: Table S5 — Association between the degree of immunodeficiency and tuberculosis (TB) with an allopatric Mycobacterium tuberculosis among European patients (n = 233) using Bayesian statistics. (PDF) [file pgen.1003318.s007.pdf]

Table S5. Association between the degree of immunodeficiency and tuberculosis with an allopatric *Mycobacterium tuberculosis* among European patients (n=233) using Bayesian statistics.

**Degree of immunodeficiency**	**Unadjusted**			**Adjusted**		
	**OR**	**95% OR**	***P* value**	**OR**	**95% OR**	***P* value**
Nadir CD4 T cell count (CD4 cells/µl)			<0.0001			0.0054
HIV-negative	1.0	(ref)		1.0	(ref)	
≥200	3.36	(0.68-16.55)		1.83	(0.34-9.94)	
50-199	4.98	(1.43-17.37)		4.11	(0.93-18.13)	
<50	8.87	(1.96-40.23)		6.65	(1.12-39.34)	

Model was adjusted for age, sex, Swiss-born, frequent travelling, contact with foreign-born population, and not HIV-associated immunosuppression (see Figure 2 for a graphical overview)

P values of linear tests for trend are shown
