# Supplementary material for: HIV Infection Disrupts the Sympatric Host–Pathogen Relationship in Human Tuberculosis
Source: PLoS Genet. 2013 Mar 7;9(3):e1003318. doi: 10.1371/journal.pgen.1003318 (PMC3591267; doi:10.1371/journal.pgen.1003318)
Supplement: Table S6 — Comparing the main phylogenetic Mycobacterium tuberculosis lineages, by HIV status and birth region. (PDF) [file pgen.1003318.s008.pdf]

Table S6. Comparing the main phylogenetic *Mycobacterium tuberculosis* lineages, by HIV status and region of birth.

Region and HIV status	No.	Main lineages, n (%)					*P* value [1]
		1	2	3	4	5+6	
**European region**	233						0.0010
HIV-infected	36	1 (2.8)	4 (11.1)	4 (11.1)	27 (75.0)	-	
HIV-negative	197	1 (0.5)	6 (3.1)	2 (1.0)	188 (95.4)	-	
**Sub-Saharan Africa (overall)**	131						0.012
HIV-infected	59	2 (3.4)	2 (3.4)	1 (1.7)	50 (84.8)	6 (8.3)	
HIV-negative	72	4 (5.6)	0 (0)	12 (16.7)	50 (69.4)	4 (6.8)	
**Eastern Africa**	55						0.12
HIV-infected	17	1 (5.9)	1 (5.9)	1 (5.9)	14 (82.4)	-	
HIV-negative	38	3 (7.9)	0 (0)	10 (26.3)	25 (65.8)	-	
**West Africa**	46						0.11
HIV-infected	30	1 (3.3)	-	-	26 (86.7)	3 (10.0)	
HIV-negative	16	1 (6.3)	-	-	10 (62.5)	5 (31.3)	
**Southern Africa**	30						0.55
HIV-infected	12	-	1 (8.3)	0 (0)	10 (83.3)	1 (8.3)	
HIV-negative	18	-	0 (0)	2 (11.1)	15 (83.3)	1 (5.6)	
**South-East Asia**	48						0.55
HIV-infected	11	7 (63.6)	4 (36.4)	-	0 (0)	-	
HIV-negative	37	18 (48.7)	14 (37.8)	-	5 (13.5)	-	
**Indian Subcontinent**	36						0.59
HIV-infected	2	1 (50.0)	0 (0)	0 (0)	1 (50.0)	-	
HIV-negative	34	8 (23.5)	3 (8.8)	13 (38.2)	10 (29.4)	-	
**Central- and South America**	24						0.13
HIV-infected	3	-	1 (33.3)	-	2 (6.7)	-	
HIV-negative	21	-	0 (0)	-	21 (100)	-	
**Middle East / North Africa**	27						0.99
HIV-infected	1	0 (0)	0 (0)	-	1 (100)	-	
HIV-negative	26	1 (3.9)	5 (19.2)	-	20 (76.9)	-	
**Western Pacific**	19						-
HIV-negative	19	-	15 (79.0)	1 (5.2)	3 (15.8)	-	

[1] Fisher's exact test

Lineage 1: Indo-Oceanic lineage; Lineage 2: East-Asian lineage (includes Beijing strains); Lineage 3: Delhi/CAS; Lineage 4: Euro-American lineage; Lineages 5 and 6: West African lineages
